# The Hospital World

**Published:** 1911-08-12

**Authors:** 


### The Hospital World.

### Women at the Museum of Surgery.

The Conservator of the Museum of the Royal College of Surgeons states that during the past collegiate year the number of visitors to the Museum was 13,343, and that more than half this number were lady visitors and nurses. The Museum is only open to women on Fridays and Saturdays, so that the attendance of women for the year is worthy of record.

### The Accountant of the North Infirmary, Cork.

Mr. Jeremiah McCarthy, who has been for over forty years accountant to the North Infirmary, Cork, has just died in Dublin. The members of the board of management, many of whom have known Mr. McCarthy for a quarter of a century, have taken the earliest opportunity of expressing their respect for his memory. Mr. McCarthy was also known locally as a warm supporter of his church, three of his sons and one daughter being regulars.

### Hospitals and the Rolandi Estate.

Foreign hospitals have a worthy share in the residuary estate of the late Mr. Frederick Rolandi, which has been left in equal shares to the Italian Hospital in Queen's Square, the French Hospital and Dispensary in Shaftesbury Avenue, the German Hospital, Dalston, the Middlesex Hospital, the Cancer Hospital, Fulham, University College Hospital, the South London Ophthalmic Hospital, St. George's Circus, and the Royal Asylum for Deaf and Dumb Poor at Margate. Mr. Rolandi left over £221,000, and the residue of the property is expected to realise more than £150,000.

### The late Sir George O'Farrell.

The recent death of Sir George Plunkett O'Farrell, who had been well-known for many years in connection with the work of prison and lunacy reforms in Ireland, at the age of 66, is a fact deplored by all interested in the treatment of mental cases. He was educated at Trinity College, Dublin, where he graduated in Medicine. He then received the diploma of the Royal College of Surgeons, England. His appointments under the Government included those of Inspector of Lunatic Asylums in Ireland, Commissioner of Prisons, Inspector of Reformatory and Industrial Schools, and Local Government Inspector. He was knighted in 1899.

### Bequests to Bootle Hospitals.

Mr. Thomas Davies, of Bootle, Liverpool, has bequeathed £1,000 to the Liverpool Royal Infirmary to endow a bed in the medical ward; £500 each to the Bootle Borough Hospital (to endow a bed in the children's ward) and the Hospital for Women, Liverpool; £250 each to St. George's Hospital for Skin Diseases, Liverpool, the Infirmary for Children, Myrtle Street, Liverpool, and to the Liverpool Bluecoat Hospital.

### The Penny Fund at Plaistow Hospital.

Miss Kate L. Ray, the Matron of St. Mary's Hospital for Women and Children, Plaistow, E., has received six thousand pennies (£25) towards her "One Million Pennies Fund," to erect a new Nurses' Home, from the Worshipful Company of Grocers. It is interesting to add in this connection that by the Matron's Million Penny Fund it is hoped also to provide a new out-patient department. The in-patients' department will shortly be transferred to a new building. About 200,000 pennies (£830) has been subscribed.

### A Governor's Bequest to Addenbrooke's Hospital.

Addenbrooke's Hospital, Cambridge, has received £1,000 from the late Mr. Philip Meyer, of Tunbridge Wells, who was for many years a governor of the hospital.

### Order of St. John of Jerusalem: Promotions.

Among the latest issue of promotions in and appointments to the Order of the Hospital of St. John of Jerusalem we note that the Hon. Surgeon-General Charles Pardey Lukis, C.S.I., Surgeon Lieutenant-Colonel Sir Warren Roland Crooke-Lawless, C.I.E., David Charles Lloyd Owen, Esq., F.R.C.S.I. (from Esquire), and Dr. Frederick Montizambert, I.S.O., have been made Knights of Grace, and as Ladies of Grace Elizabeth Mrs. E. F. Chinery (from Honorary Associate), the Hon. Lady Harvey, the Hon. Lady Haig, Miss Helena Constance Frank, Miss Mary Harriett Griffith, her Excellency Lady Clarke, the Lady Catherine Milnes-Gaskell. John Horatio Yolland, Esq., M.R.C.S., L.S.A. (from Honorary Associate), Major Gilbert Stewart Crawford, R.A.M.C., M.D. (from Honorary Associate), and Thomas Gibson, Esq., M.B., C.M., have been made Esquires.

### Gifts in Kind for Addenbrooke's Hospital.

As new-laid eggs have been somewhat plentiful this season, the authorities of Addenbrooke's Hospital, Cambridge, have circularised the headmasters and headmistresses of the various schools in a large portion of the district served by the hospital asking them whether they could very kindly organise egg collections for the benefit of the hospital. The appeal met with such generous response that Addenbrooke's has received during the past quarter no fewer than 9,000 new-laid eggs, which have assisted the funds greatly. In most instances the masters, mistresses, and scholars have expressed a wish to make a similar collection annually. Moreover, for the past three years collections of gifts in kind have been organised in some of the villages in the district, with the most satisfactory result. The following is a typical broadside, and explains how the collections are arranged: "Addenbrooke's Hospital, Cambridge. A special appeal will be made to the residents of villages, which are benefited by the hospital, for gifts of fruit, vegetables, potatoes, honey, poultry, eggs, butter, meat, tea, etc., which are at all times most acceptable, and not only afford a welcome change in the daily dietary, but materially assist the funds. It has been suggested that a collection of gifts in kind would be heartily supported in [here occurs the name of the village] district, during the week from [and here the date]. The hospital seldom receives an excessive amount of such gifts in kind, and when it does the gifts are carefully stored. The stock of potatoes, onions, carrots, etc., received last autumn has just come to an end." The broadside concludes with the name and address of the receiver of the gifts, which is often that of the local postmaster.

